# Exploring near-infrared absorbing nanocarriers to overcome cancer drug resistance

**DOI:** 10.20517/cdr.2020.20

**Published:** 2020-07-02

**Authors:** Siwei Chu, Ursula Stochaj

**Affiliations:** Department of Physiology, Faculty of Medicine, McGill University, Montreal H3G1Y6, Quebec, Canada.

**Keywords:** Cancer drug resistance, chemotherapy, nanomedicine, near-infrared light, combination therapy, photothermal therapy, photodynamic therapy, controlled drug release

## Abstract

One of the major obstacles of successful cancer therapy is cancer drug resistance. The unique tools and applications developed by nanomedicine provide new approaches to surmount this common limitation of current treatment regimens. Nanocarriers that absorb light in the near-infrared spectrum are particularly suitable for this purpose. These nanocarriers can produce heat, release drugs or stimulate the production of physiologically relevant compounds when illuminated with near-infrared light. The current review summarizes the causes contributing to cancer multidrug resistance. The major types of nanocarriers that have been developed in recent years to overcome these hurdles are described. We focus on nanoparticles that are responsive to near-infrared light and suitable to surmount cancer multidrug resistance. Our review concludes with the bottlenecks that currently restrict the use of nanocarriers in the clinic and an outlook on future directions.

## Introduction

### Drug resistance, a persistent bottleneck for cancer therapy

At present, surgery, radiation and chemotherapy are the leading regimens for cancer treatment which are often combined with targeted or immunotherapy^[1]^. The advantages of chemotherapy include highly standardized treatment protocols, and extensive experience has been gained with their clinical applications. However, drug-resistant cancer cells can be inherent to the cancer or are acquired during therapy. Multidrug resistance (MDR) in particular compromises treatment efficacy and promotes disease recurrence and cancer-related patient death^[2]^. MDR has been defined as the “resistance of cancer cells to structurally and mechanistically unrelated classes of anticancer drugs”^[3]^. Treatment resistance is not limited to chemotherapy, but also applies to targeted and immunotherapy^[4]^. Dose escalation has been used to counter drug resistance, but it fails to eliminate all tumor cells^[5]^. Moreover, the increased concentration of cancer drugs is often associated with severe side effects. While elevated dose intensity regimens limit the recurrence of some cancers^[5]^, alternative or complementary strategies are needed to combat cancer drug resistance. Nanocarriers are especially promising tools for the development of such complementary approaches. To maximize the efficacy of nanocarriers, it is important to consider the different origins of cancer drug resistance.

### Mechanisms of cancer drug resistance

The causes of treatment resistance are not limited to the tumor cells. They are often multifactorial^[2]^ and include the complex tumor microenvironment (TME). The heterogeneity of cancer cells within the tumor, temporal changes in tumor cell properties, and differences among metastases complicate the design of proper treatment regimens.

Antineoplastic resistance may be intrinsic to the tumor or develop during therapy. A network of factors collaborates to promote treatment resistance; such resistance can be obtained through pathway reactivation, bypass or indifference^[2]^. Cancer cells acquire drug resistance through mutations in key signaling molecules^[6]^ or non-genetic changes^[7]^. Chemoresistance is linked to diverse targets that control drug transport, signaling and metabolic routes; they determine cancer cell survival and proliferation^[2,8]^. Notably, a subset of these targets is non-druggable (see Future Directions). Non-druggable and particularly important to therapy are mutations in genes encoding oncogenic drivers, as exemplified by *MYC*, *RAS*, *TP53*, and *BRAF*^[9]^. In addition, cancer stem cells (CSCs), a subpopulation of tumor cells with self-renewal capabilities, often display greatly elevated treatment resistance^[10,11]^.

Cancer cell and CSC survival is promoted by reduced intracellular drug concentrations, suppressed apoptosis, enhanced damage repair, and changes in the abundance of drug targets^[11-13]^. Changes in drug transport and metabolism represent major paths to chemotherapeutic failure^[12]^. Specific mechanisms contributing to MDR include the activation of anti-apoptotic signaling, inactivation of pro-apoptotic signals, environment-mediated drug resistance^[14]^, cell dormancy^[15]^, altered microRNA profiles, and the production of extracellular vesicles^[16]^. In the context of near-infrared (NIR) absorbing nanocarriers, research has focused mainly on intracellular drug concentrations, which are determined by drug uptake, metabolism and efflux.

#### ABC transporters

Cancer drug efflux mediated by the ATP-binding cassette (ABC) transporter family is especially well understood^[3,17,18]^. ABC family members move structurally diverse compounds across cellular membranes. Among the ABC transporter substrates are agents that impinge on various cancer survival pathways. Besides common chemotherapeutic agents, several ABC transporters also mediate the efflux of compounds used for molecularly targeted therapy^[19]^.

The overexpression of several ABC transporters stimulates cancer drug efflux through an ATP-dependent process^[3]^. Notably, overabundant ABC transporters promote MDR in tumor cells; their overexpression can be particularly high in CSCs^[15]^. Various ABC transporters are critical contributors to MDR, especially members of the ABCB, ABCC, and ABCG subfamily^[3,17,20]^. Several key players have been identified: (1) permeability-associated glycoprotein (P-gp, P-glycoprotein, ABCB1, encoded by the *MDR1* gene); (2) multidrug resistance-associated protein 1 (MRP1, ABCC1); (3) breast cancer resistance protein (BCRP, ABCG2); and (4) MRP2 (ABCC2).

#### P-Glycoprotein

To date, P-gp is one of the most common host factors for cancer MDR that has been targeted with nanoparticles (NPs). Like other ABC transporters, P-gp is an integral membrane protein that is critical for the transport of diverse cargos across cell membranes^[21]^. P-gp is particularly abundant in the plasma membrane of cells that excrete physiological substrates and xenobiotics^[22]^. P-gp resides in epithelial cells of the intestine, renal proximal tubules, liver bile ductules, and other cell types^[3,17,22]^. P-gp cargos include a wide spectrum of endogenous substrates, such as hormones, cytokines, chemokines, and nutrients^[23]^. Moreover, P-gp transports neutral and cationic hydrophobic compounds that are used in the clinic. Specific examples are taxanes (such as docetaxel), anthracyclines (doxorubicin), antibiotics, and tyrosine kinase inhibitors^[3,17,24]^. Thus, P-gp affects the pharmacokinetics of numerous drugs^[3,17,24]^.

P-gp activities have multiple consequences for therapy. P-gp (1) restricts the intestinal absorption of drugs, (2) controls drug passage through the blood-brain-barrier, and (3) promotes drug excretion through renal and hepatobiliary routes^[25]^. Hence, P-gp activities can profoundly diminish the cytotoxicity of therapeutic agents. Taken together, P-gp regulates the systemic concentration of many chemotherapeutic compounds and thereby often contributes to MDR.

The organization and molecular functions of P-gp have been studied extensively^[24,26]^. In brief, alternative splicing and single nucleotide polymorphisms produce several P-gp isoforms. Human P-gp consists of 1276-1280 amino acid residues which are organized into two repeating units. Each individual unit contains a cytoplasmic nucleotide-binding domain (binds ATP) and a transmembrane domain. The two units collaborate in the P-gp transport cycle. While the transmembrane domains bind the substrate, the nucleotide-binding domains are required for ATP binding and hydrolysis^[26]^.

Several strategies have been explored to control P-gp activity. This can be achieved by modulating P-gp (1) substrate binding and transport, (2) ATPase cycle, (3) membrane environment, (4) protein stability, (5) gene expression, and (6) mRNA translation. To date, different generations of ABC transporter inhibitors have been synthesized and evaluated. However, they have limited efficiency^[17]^ and often display off-target effects^[27]^. As these compounds are unlikely to overcome MDR, alternative strategies are being developed. Several of these strategies rely on nanocarriers that respond to NIR light.

#### Other molecular factors promoting cancer multidrug resistance

Aside from ABC transporters, additional cellular factors facilitate MDR through mechanisms that are driven by protein-protein interactions or gene regulatory networks^[28-30]^. For example, transcriptional regulators and epigenetic mechanisms determine *de novo* gene expression, which can ultimately enhance MDR. As such, the transcription factor NF-κB and mutant p53 control *MDR1* gene expression and thereby modulate the response to chemotherapeutic drugs. Notably, both NF-κB and mutant p53 are targetable with NIR-responsive NPs^[31]^ (see section on gold nanoparticles). Other factors linked to MDR include target mutations, drug metabolism, increase in repair enzymes, antioxidant components, and molecular chaperones^[30,32]^. Regulated in part by the TME, pH, oxygen, and nutrient supply of the cancer cells also shape the response to chemotherapeutic agents^[16]^. For example, hypoxia-inducible transcription factors (HIF-1, HIF-2) can upregulate the expression of genes encoding drug efflux pumps^[16]^. At present, several components relevant to MDR have yet to be incorporated in NP-based strategies (see Future Directions).

#### Resistance to doxorubicin, cisplatin or docetaxel

**Figure 1:**
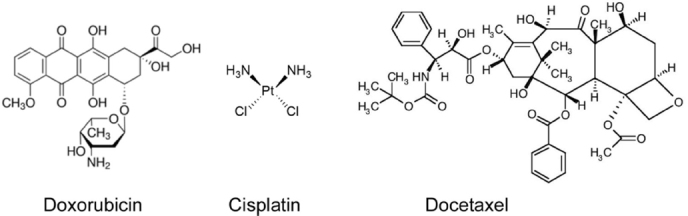
Chemical structures of doxorubicin, cisplatin, and docetaxel

Many of the studies related to cancer MDR and nanomedicine concentrate on the resistance to doxorubicin^[33-35]^, cisplatin^[34,36,37]^, or docetaxel^[38-42]^. These drugs are frequently used for research on stimulus-responsive NPs. In the clinic, the anthracycline doxorubicin [Figure 1] is applied to treat a variety of malignancies^[35]^. Several biological processes are associated with the resistance to anthracyclines, including drug transport, topoisomerase II activity, DNA repair, cancer stemness, and drug metabolism^[35]^. In the context of stimulus-responsive NPs, doxorubicin-related studies have focused predominantly on drug efflux. Several ABC transporters are implicated in doxorubicin efflux, such as P-gp (ABCB1), MRP1 (ABCC1) and BCRP (ABCG2)^[3,35]^. Depending on the type of cancer, additional carriers may add to doxorubicin resistance^[35]^.

A large number of cancers are treated with cisplatin. Cisplatin resistance is determined by multiple factors that fall into three major categories: DNA repair, intracellular accumulation, and drug modifications in the cytoplasm^[37]^. Various membrane transporters participate in the uptake and efflux of cisplatin and additional platinum compounds^[37,43-45]^. The copper transporters CRT1 and CTR2, as well as members of the organic cation transporter family (OCT) facilitate cisplatin uptake^[37,43]^. MRP2 and other transmembrane proteins have been linked to cisplatin efflux^[34,37]^.

Microtubule stabilizers and destabilizers are widely used for the treatment of malignant solid tumors^[38]^. Docetaxel is a taxane that binds β-tubulin, which stabilizes microtubules and prevents the completion of cell division^[38-42]^. Several mechanisms contribute to docetaxel resistance. These include mostly efflux through P-gp and other ABC transporters, mutation of the drug-binding site of β-tubulin, and the synthesis of an alternative β-tubulin isoform^[3,38,42]^. Further routes to docetaxel resistance have been identified^[42]^.

#### Strategies to overcome drug resistance in the clinic

The complexity and heterogeneity of tumors, even within the same patient, pose major obstacles for the selection of appropriate treatment regimens. Precision medicine applies the genetic information on the patient’s cancer and pharmacogenomics to optimize targeted treatment^[16,46-49]^. This bio-guided approach relies on genetic information on molecular markers to block oncogenic pathways^[48]^. Specifically, identifying genetic variants of oncogenes or tumor suppressors aids in selection of the most appropriate chemotherapy for each patient. This is crucial as the response to drug treatment is highly variable and to a large extent driven by genetic variants^[16]^. Pharmacogenomics Knowledgebase (PharmGKB^[50]^) provides information on drug-gene interactions and thus helps to identify the most suitable approach for targeted therapy.

### Nanomedicine for cancer therapy

The unique properties of nanomaterials have encouraged the development of nano-based theranostics. Drug delivery through nano-sized carriers can lead to marked changes in whole-body distribution, pharmacokinetics, and pharmacodynamics of the agent. Patients may further benefit from the intrinsic therapeutic properties of some nanomaterials^[51]^. Numerous nanomaterials are currently evaluated for health applications^[52]^. In particular, NPs with defined composition, size, morphology (e.g., spheres, hexagons, rods, sheets), and surface coating are being assessed for diagnosis and therapy. As discussed in the following section, NP-based nanomedicine provides distinctive advantages that are not always available through other applications.

#### Extending drug circulation time and bypassing drug efflux

A major rationale for the development of therapeutic NPs is that NP-mediated drug delivery can reduce the treatment-associated toxicity. Indeed, NP-assisted drug delivery offers several therapeutic benefits, especially in the context of cancer drug resistance. First, free anti-cancer drugs often have a short systemic circulation time. Before reaching the desired sites, they may be removed from the circulation, mainly through interstitial diffusion, non-specific binding, glomerular filtration, as well as hepatic clearance^[53]^. At the cellular level, drug metabolism and efflux are major contributors to drug clearance. However, when protected by NP association, chemotherapeutic drugs can maintain a higher concentration and prolonged residence in the circulatory system^[54]^.

Second, drug efflux opposes the accumulation of free drugs in cancer cells. It is typically mediated by transporters located in the plasma membrane (see above). By contrast, cells often internalize drug-NP complexes through endocytic pathways. These routes differ fundamentally from transporter-mediated uptake, as they include clathrin- or caveolae-mediated endocytosis and rely on vesicle trafficking^[55]^.

Enclosed in endosomal vesicles, NP-associated drugs are protected against efflux from the cell. Moreover, during vesicle trafficking, drug release can be accomplished at subcellular locations where efflux pumps are sparse. Together, these mechanisms increase intracellular drug concentrations. Importantly, NP-based drug formulations often reduce side effects, which improves patient adherence to therapy and thus overall treatment success. Targeting NPs to desired locations (see next section) can further improve NP-based therapy.

#### Targeted drug delivery

NPs can target tumors and the TME through passive or active processes^[56]^. Various approaches have been explored to enhance NP accumulation at these sites. However, it should be emphasized that improved NP delivery to the tumor or TME is only meaningful in the clinic if it increases treatment success^[57,58]^. Enhanced permeability and retention (EPR) has been described as the main basis for passive targeting to solid tumors, but the importance of EPR for the treatment of human tumors continues to be debated^[57,58]^. A recent study points to alternative mechanisms and highlights the role of transcytosis across endothelial cells for NP extravasation into the tumor^[59]^. Active tumor targeting has been accomplished by various NP modifications. Conjugation to antibodies, ligands, or aptamers has directed NPs to the surface of malignant cells or to the TME^[56,60,61]^.

Both passive and active tumor targeting strategies can enhance drug supply to the tumor site, which affords important therapeutic benefits. First, drug concentrations increase in cancer cells. Second, with elevated concentrations in the tumor, systemic drug doses can be diminished. This in turn may reduce side effects. A recent phase I trial in human patients with solid tumors demonstrated the benefits of NP-mediated drug delivery^[62]^. Compared with the free drug (docetaxel), docetaxel-loaded NPs increased intratumoral drug concentrations, while significantly diminishing adverse outcomes^[63]^.

### Stimulus-responsive nanoparticles for cancer therapy

Nanomaterials that respond to specific stimuli are interesting for therapy as their functionalities can be controlled by specific cues. As a result, stimulus-responsive NPs offer excellent spatiotemporal control of their functionalities. Such control can be achieved through internal or external cues [Table 1]^[58,64]^. For example, pH, redox state, and enzymatic activities of subcellular organelles, tissues or organs can provide internal triggers for NP-dependent actions. These parameters are relevant to cancer therapy as tumor cells and the TME are generally more acidic. They also exhibit altered redox properties and enzymatic activities^[64-66]^.

**Table 1:** Control of nanoparticle functionalities

Stimulus	Internal (provided by cells, tissues, extracellular milieu)	External
Light	NA	Natural light exposure of skin, retina; targeted light exposure via laser or other artificial light sources; different wavelengths (UV, visible, NIR); photo-acoustic therapy
pH	Tumor microenvironment^[65]^; tissues, organs, biological fluids: stomach (pH 1.5-3.5), intestine (slightly alkaline), blood (pH ~7.3-7.5), lymph (slightly alkaline); subcellular organelles with low pH: lysosomes, endosomes, Golgi apparatus (pH 4.0-6.5)^[67]^	NA
Redox environment	Endoplasmic reticulum (oxidative protein folding^[68]^); TME may increase oxidative stress in tumor cells^[65]^	NA
Heat	Body temperature	Extrinsic trigger to change temperature of biological environment; laser irradiation at different wavelengths^[69]^; alternating magnetic fields (induction)^[70]^
Cold	NA	Cold-induced drug release^[71]^; cryosurgery^[72]^
Ultrasound	NA	Disintegration of NP clusters^[73]^
Metabolites	Glutathione, increased concentrations in tumor cells; lactate, elevated concentrations in tumor and TME	NA
Magnet	NA	External magnetic field

Stimuli that have been used to regulate nanoparticle functionalities are depicted. The list is not comprehensive; it focuses on triggers that are well-established for nanomedical approaches. NIR: near-infrared; TME: tumor microenvironment

The extrinsic control of NP performance differs in several aspects from intrinsic regulation. Extrinsic triggers can limit off-target effects in healthy tissues and have the potential for precise eradication of malignant cells^[58]^. However, this requires that tumor size and location are known, which is difficult if tumors or metastases are small or spread over multiple anatomical locations. Thus, NPs controlled by external stimuli will be particularly useful to treat localized tumors that are not resectable.

External triggers, especially light, have been employed to regulate diverse NP functionalities. Relevant to cancer treatment are NP-based drug sequestration, stabilization, and delivery. In addition, the NP-mediated heat or ROS production and enzyme activation have also been explored [Table 1].

#### Light-responsive nanoparticles

Wavelengths throughout the electromagnetic spectrum have been employed to control stimulus-dependent NPs^[74,75]^. However, there are restrictions for *in vivo* applications, because UV and visible light is strongly absorbed by biological material^[75,76]^. Possible consequences are damage to biomolecules, elevated tissue temperature, and light scattering accompanied by reduced tissue penetration^[77-79]^. By contrast, NIR irradiation can achieve deeper tissue penetration^[75,79-81]^. The NIR window between 700 nm and 1,000 nm is ideal for light-mediated therapeutics. Depending on the power density and properties of the biological material, NIR may penetrate to a depth of 3 cm^[75,82]^. This value may be limited to unique conditions, as many publications suggest a penetration depth of 1 cm or less^[79,81]^. Crucial for therapy, NIR exposure is associated with low toxicity for healthy tissues^[83]^. Although NIR laser light can be phototoxic at high doses, genotoxic and mutagenic effects are likely minor^[84]^. Nevertheless, at high laser irradiance, NIR may increase the local temperature of biological specimens and produce reactive oxygen species (ROS)^[84]^. On the other hand, this heat and ROS production can be exploited for tumor cell eradication. Indeed, both modalities have been incorporated in nanotherapeutic regimens (see below).

### Therapeutic applications of stimulus-responsive nanoparticles

**Figure 2:**
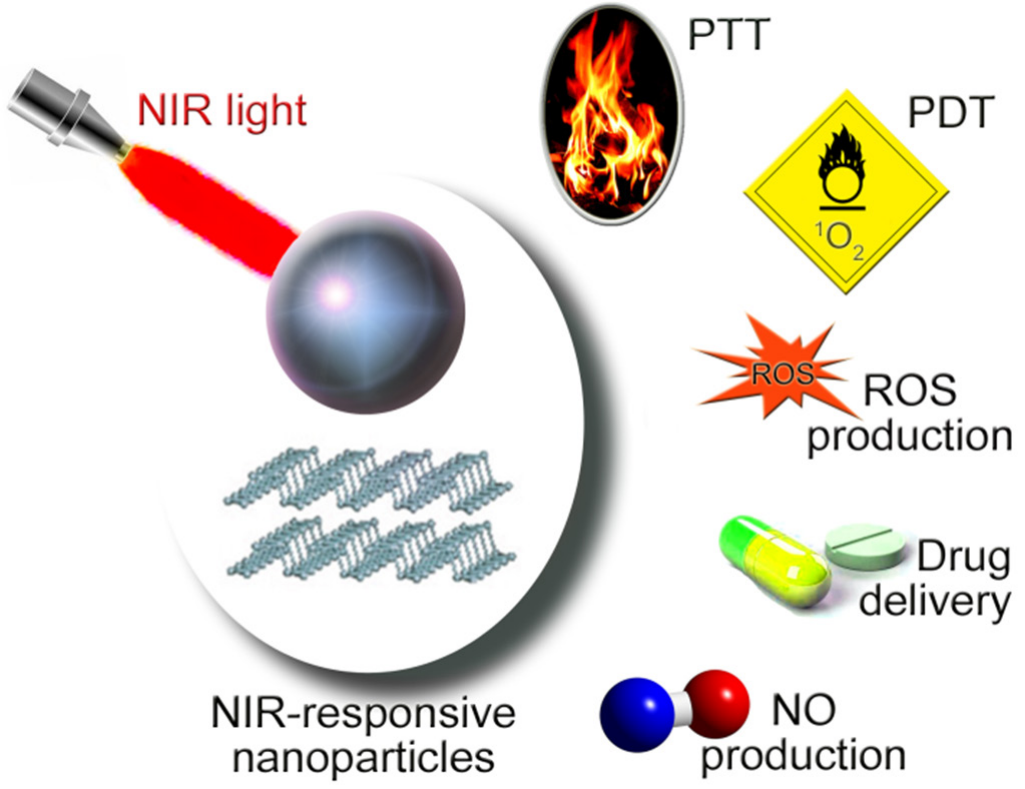
NIR-responsive NP actions relevant to cancer MDR. NIR-responsive NPs have been evaluated for their ability to surmount the drug resistance of cancer cells. Spherical NPs and nanosheets are depicted as examples. A set of NP-based treatment modalities has been explored *in vitro* and *in vivo*. PTT, PDT, ROS production, drug delivery, and NO production are the main applications that have been examined to date. NIR: near-infrared; NP: nanoparticle; MDR: Multidrug resistance; PTT: photothermal therapy; PDT: photodynamic therapy; ROS: reactive oxygen species; NO: nitric oxide

This section of the review focuses on NIR as an external trigger to activate NP-based activities. Figure 2 illustrates the major functionalities of NIR-responsive NPs that have been assessed for the elimination of MDR cancer cells.

#### Photothermal therapy

Photothermal therapy (PTT) is a minimally invasive treatment modality that locally elevates cell or tissue temperature. Several molecular mechanisms make cancer cells more sensitive to heat than their normal counterparts^[85,86]^. These differences are clinically relevant and have been exploited for PTT. Specifically, localized heat generation destroys cancer cells, which may include drug-resistant tumor cells. The most common modes of PTT-induced death are apoptosis and necrosis. While photothermal ablation of the tumor is often incomplete^[87]^, heat is nevertheless beneficial to combat MDR. It may re-sensitize resistant cancer cells to chemotherapy through different pathways. These routes include up-regulating the abundance of heat shock factor (HSF-1) or decreasing P-gp levels^[88]^.

Depending on the nanomaterial properties, the intensity and duration of irradiation, NIR light can increase NP temperatures to 40-50 °C and above^[87,89,90]^. NIR-absorbing organic small molecules can also serve as sensitizers for PTT. When incorporated in nanocarriers they can provide or enhance PTT modalities^[91]^. The nanomaterials discussed here differ widely in their ability to generate and withstand heat. For example, platinum-containing NPs have extreme heat stability^[92]^. However, it is not clear whether this is relevant to clinical applications, especially since temperatures > 100 °C reduce the efficiency of thermal tumor ablation^[85]^.

#### Photodynamic therapy

Photodynamic therapy (PDT) exploits the cytotoxicity of reactive molecular species (ROS; reactive nitrogen species, RNS). The treatment requires the administration of a photosensitizer, which absorbs light at a defined wavelength, a light source, and oxygen (or another substrate)^[93-95]^. Following irradiation, elevated levels of reactive molecular species have twofold impact on the tumor; they may kill cancer cells directly, or affect the TME, especially tumor vessels. The major routes of cancer cell death caused by PDT include apoptosis, necrosis, autophagy-dependent death, and paraptosis^[94,95]^. Interestingly, the PDT-induced ROS production may damage some of the ABC transporters, which can diminish MDR^[96]^. Like thermal ablation^[85]^, PDT has been associated with enhanced adaptive immunity or immunosuppression^[94]^. A key step for PDT is the efficient delivery of photosensitizers to the tumor sites. Photosensitizers are often hydrophobic and prone to aggregation, but their delivery by nanocarriers can circumvent this difficulty^[95]^.

#### Controlled drug release

NIR light can trigger drug release from nanocarriers in a confined space and highly regulated temporal fashion. The strategy of controlled drug release (CDR)^[97]^ elevates drug concentrations at tumor sites, without a concomitant overall increase in the circulatory system. The localized rise in drug concentration at the tumor sites is advantageous in the context of MDR, as it could exceed the capacity of efflux pumps. A potential outcome is the killing of chemoresistant cancer cells. Moreover, the drug-related side effects will be limited, as healthy tissues and organs have only limited exposure to chemotherapeutic agents.

#### Nitric oxide generation

Doxorubicin, a commonly used chemotherapy drug, stimulates nitric oxide (NO) production, in part through the increase in NO synthase activity^[98]^. NO synthesis contributes to the cytotoxicity of doxorubicin^[98]^, but is diminished in doxorubicin-resistant cells^[99]^. Notably, increased NO concentrations impair drug efflux pumps^[99]^, which reduces MDR. The combination of NO donors with NIR-responsive nanocarriers exploits this effect and facilitates drug accumulation in MDR cancer cells.

#### Combination therapy

In combination therapy, two or more treatment modalities are used together to improve treatment success. These modalities may target different cellular mechanisms that contribute to cancer cell survival and proliferation^[100]^. By hitting multiple targets, the simultaneous modulation of several signaling pathways or synthetic-lethal strategies increase the likelihood of cancer cell killing^[100]^.

NP-based combination therapy offers advantages over other approaches. For example, when multiple drugs are combined, differences in the pharmacokinetics may prevent optimal treatment outcome for drug combinations^[101]^. Nanomedicine may surmount this hurdle, as NP-based co-encapsulation supports synchronized drug delivery to the tumor. Furthermore, PDT-induced killing of cancer cells can be enhanced when photosensitizers are loaded onto NPs and released in a localized manner^[94]^. NP-based combination therapy is also promising to combat cancer drug resistance. To achieve this, NPs have served as carriers for efflux pump inhibitors^[29,102,103]^, siRNAs, pro-apoptotic molecules, therapeutic antibodies, and other agents^[104,105]^. The combination of thermal ablation with other therapeutic modalities is especially promising for NP-dependent approaches^[106]^.

## Near-infrared light absorbing nanomaterials for cancer therapy

A variety of nanomaterials with strong NIR absorption have been developed and tested in recent years. Depending on the selection of NPs and settings for NIR irradiation, different mechanisms contribute to light-induced drug release. On the level of NPs they include NIR-triggered disintegration, destruction of drug-carrier bonds, and removal of ‘gate-keepers’ from mesoporous NPs^[107]^. The use of pulsed *vs.* continuous wave NIR also determines the impact on cancer cell viability, which has been attributed in part to differences in drug release^[108]^. By combining the advantages of NP-assisted drug delivery and light-controlled drug release with PTT or PDT, NIR-absorbing NPs have great potential to defeat cancer drug resistance. In the following section, we briefly describe *in vitro* and *in vivo* models that are commonly used for nanomedical research in the context of drug resistance [Table 2]. NIR-absorbing nanocarriers are then introduced as tools for cancer therapy and specific examples discuss NP-based approaches to overcome MDR.

**Table 2:** Frequently used cell lines and compounds to evaluate NIR-responsive nanoparticles in the context of multidrug resistance

Cell line, targeting moieties, drugs, other reagents	Properties, references
“MCF-7/AD”, re-classified as NCI/ADR-RES; 2D cultures and 3D spheroids; also used in different mouse models	Doxorubicin-resistant; derived from human ovarian carcinoma (OVCAR-8) cells^[109,110]^; MDR^[88,111-129]^
SW620/Ad300 cells	Doxorubicin-resistant; obtained from human colorectal adenocarcinoma cells SW620; MDR^[102,130]^
HT29-dox cells	Doxorubicin-resistant; obtained from human colon epithelial cells HT29; MDR^[99]^
A2780cisR cells	Human ovarian carcinoma; cisplatin-resistant; MDR^[131,132]^
SCC-7 cells	Mouse, squamous cell carcinoma cells^[111]^
COS7 cells	African green monkey, kidney fibroblasts^[111]^
4T1 cells	Mouse, mammary gland carcinoma cells^[114]^
HeLa cells	Human, cervix adenocarcinoma^[114]^
MDA-MB-231 cells	Human, mammary adenocarcinoma; chemoresistant; overexpression of several ATP transporters^[133,134]^
MDA-MB-453 cells	Human mammary gland, metastatic carcinoma; high DNA methyltransferase (DNMT) activity; chemoresistant; hypermethylator phenotype; mutant phosphatase *PTEN* gene^[135-137]^
HepG2cisR cells	Human, hepatocellular carcinoma; cisplatin-resistant, MDR^[132]^
K562R cells	Human, chronic myelogenous leukemia; elevated abundance of P-gp; MDR^[138,139]^
Hyaluronic acid (HA); used for cell targeting	Natural glycosaminoglycan polysaccharide; binds to CD44 receptor on cell surface; biodegradable by hyaluronidases^[140-142]^
Folate; used for cell targeting	Vitamin B9, also called folic acid; binds to folate receptor on cell surface^[143,144]^
Transferrin	Iron-binding protein recognized by transferrin receptor (TfR); TfR located on cell surface; TfR abundance often highly increased in cancer cells^[145]^
Doxorubicin	Multiple modes of action, such as formation of DNA adducts, inhibition of topoisomerase II, DNA and RNA polymerases; also called adriamycin^[33,35]^
Quercetin	Natural polyphenolic flavonoid present in fruits and vegetables; may have chemopreventive activity^[146]^
3-Methyl adenine	Inhibits autophagy^[147]^
Chloroquine	Inhibits autophagy^[148]^
D-α-Tocopherol polyethylene glycol succinate	P-gp inhibitor^[149]^, also called tocophersolan, TPGS
Tetradecanol	Long-chain fatty alcohol^[150]^, undergoes phase change
Cisplatin	Produces DNA lesions^[36,37]^
Irinotecan	Also called camptothecin-11 or CPT-11; activated by carboxylase-converting enzyme, which generates SN-38; SN-38 inhibits topoisomerase I^[151,152]^
Docetaxel	Binds and stabilizes microtubules^[39-41]^
Poloxamer 188	Ethylene oxide/propylene oxide copolymer; likely inhibits P-gp^[133,153,154]^
Polyethylene glycol (PEG)	Polymer; used for NP coating^[155]^

Multidrug resistance (MDR) has been demonstrated where indicated. NP: nanoparticle; TPGS: D-α-tocopherol polyethylene glycol succinate; NIR: near-infrared

### Cellular and animal model systems to study cancer MDR

Different cellular and animal models have been developed to investigate tumor cell MDR. Model systems to examine transporter-dependent drug efflux have been reviewed recently^[156]^. Many studies on NIR-responsive NPs focus on efflux pumps. Several reagents and models are commonly employed by different research groups. They are briefly described here and listed in Table 2.

#### Cell lines and spheroids

A large number of studies relied on “MCF-7/ADR” cells. Initially considered a derivative of the human epithelial breast cancer cell line MCF-7, the cells have been re-classified as NCI/ADR-RES^[109]^. It was demonstrated that this cell line originates from human ovarian carcinoma (OVCAR-8) cells. Unlike MCF-7 cells, NCI/ADR-RES cells are caspase-3 positive and carry a rare p53 mutation. However, NCI/ADR-RES cells do exhibit doxorubicin-resistance, which is crucial to the studies discussed here. For simplicity, we adhere to the nomenclature used in the original publications, but refer to the cells as “MCF-7/ADR” to indicate their re-classification. Other common MDR model systems are SW620/Ad300 and HT29-dox cells. The human colorectal adenocarcinoma cell line SW620 was used to generate the drug resistant sub-line SW620/Ad300^[130]^. HT29-dox is a doxorubicin-resistant derivative of HT29 cells, a human colon epithelial cell line^[99]^. Additional cell lines relevant here are listed in Table 2. Aside from 2D cultures, NIR-responsive NPs are also assessed with 3D spheroid models of MDR^[157]^. As spheroids mimic multiple *in vivo* properties of solid tumors, they are especially valuable to assess NPs and other anti-cancer agents^[158-160]^. In general, cancer spheroids are more resistant to treatment when compared with 2D cultures^[158-160]^. Several parameters have been identified that determine NP penetration of tumor spheroids^[161]^.

#### Experimental animals

Some studies on MDR and NIR-responsive NPs have been carried out with experimental animals *in vivo*. In most cases, the research involved mice and heterotopic xenograft models. Tumor cells were often injected subcutaneously or into the tail vein.

#### Anticancer drugs and other relevant agents

Many of the NP-based research described here has been performed with the chemotherapeutic agents doxorubicin and cisplatin, whereas the use of docetaxel was less common [Figure 1]. The anthracycline doxorubicin targets DNA topoisomerase II^[162]^ and has multiple synonyms, such as adriamycin^[33,35]^. Cisplatin and its derivatives cause DNA damage^[36,37]^; the taxane docetaxel stabilizes microtubules^[38-42]^. Some studies also included inhibitors of autophagy or natural compounds [Table 2].

Recent work incorporated phase change material in NIR-responsive NPs^[112]^ to promote the temperature-dependent release of pharmacological agents. For example, 1-tetradecanol has a melting point around 38-39 °C, which is ideal for the NIR-induced discharge of pharmacological compounds.

## Surmounting cancer MDR with NIR-responsive nanoparticles

The impact of various NIR-responsive NPs has been examined in drug-resistant cancer cells. The NPs belong to different groups [Table 3]. They have been evaluated *in vitro* and *in vivo*; specific examples are described in the following sections and listed in Table 4.

**Table 3:** NIR-absorbing nanomaterials with potential for NP-based health applications

Nanoparticle group	Nanoparticle subgroups	Ref.
Noble metals	Gold, silver, platinum, palladium	[88,102,111,113,114,163,164]
Lanthanides	Upconverting NPs	[115,123,126,131,165]
Metal chalcogenides	Copper chalcogenides, transition metal dichalcogenides	[117,166-170]
Metal oxides	Iron oxide, titanium oxide, tungsten oxide	[157,171]
Carbon	Carbon nanotubes, carbon nanospheres, graphene oxides	[124,125]
Polymers	Polydopamine	[120,127-129]
Black phosphorus	NA	[120,132]

Near-infrared (NIR)-responsive nanomaterials listed are pertinent to the treatment of cancer multidrug resistance (MDR) and discussed in this review. References indicate successful applications, using *in vitro* and/or *in vivo* MDR models. Some composite nanoparticles (NPs) fall into multiple classes

**Table 4:** Representative NIR-absorbing nanocarriers used to overcome cancer drug resistance. The references provide a detailed composition of the nanoplatforms

Category	Platform	Application	Drug	NIR (nm)	Ref.
Noble metals	AuNS-pep-HA	PTT	DOX	808	[111]
	AuNR-CuS-Liposome	NO generation	DOX	808	[238]
	PLGA-AuHS-death receptor4	PTT	DOX	808	[31]
	TD-AuNC-PEG-Biotin	PDT, CDR	DOX, QUR	808	[112]
	AuNP-SiO_2_	PTT	DOX	780	[88]
	AuNR-mSiO_2_-PHIS-TPGS	PTT, CDR	DOX	808	[102]
	PtNP-Fucoidan	PTT	DOX	808	[113]
	CuPd	PTT	DOX, CQ, 3-MA	808	[114]
Lanthanides	NaYF_4_:Yb/Tm-TiO_2_-FA	PDT	DOX	980	[115]
	NaGdF_4_:Yb/Nd-NaGdF_4_:Yb/Er-NaGdF_4_	PDT	RB, Pt(IV) prodrug	808	[131]
	NaGdF_4_:Tm/Yb-NaGdF_4_-OA-Azo-Lipo	CDR	DOX	980	[123]
Copper chalcogenides	CuS-mSiO_2_-SNO	NO generation	DOX	808	[116]
	Cu_2-x_Te NC-PEG	PTT, PDT	DOX	808	[136]
Transition metal dichalcogenides	MoS_2_-PEI-HA	PTT	DOX	808	[117]
Metal oxides	Fe_3_O_4_-PDA-mSiO_2_	NO generation	DOX	808	[118]
Carbon-based	Graphene oxide-PEG-DA	PTT	DOX	808	[119]
	Graphene oxide	PTT	DOX, irinotecan	808	[133]
	Single-walled carbon nanotubes-Ap	PTT	DOX	ns	[138]
	Single-walled carbon nanotubes	PTT	DOX	808	[125]
	Hollow carbon nanospheres	PTT, CDR	DOX	780	[124]
Polydopamine	PLGA-PDA-TPGS	PTT, CDR	DTX	808	[129]
	PDA-PEG-FA	PTT, CDR	DOX, BNN6	808	[128]
	PNOC-PDA	PTT, CDR	DOX	808	[127]
Black phosphorus	BP-PDA-PEG-Apt	PTT, CDR	DOX	808	[120]
	Pt-BP	PTT	Cisplatin	808	[132]
	BP-PDA-PEG-PEITC	PTT, PDT	DOX	808	[121]

Near-infrared (NIR) refers to the wavelength that was used to trigger a nanoparticle (NP) response. NO: nitric oxide; CDR: controlled drug release; CQ: chloroquine; DA: 2,3-dimethylmaleic anhydride; DOX: doxorubicin; 3-MA: 3-methyl adenine; ns: not specified; PDT: photodynamic therapy; PTT: photothermal therapy; QUR: quercetin; RB: rose bengal

### Localized surface plasmon resonance

Noble metal nanostructures, such as gold (AuNPs), silver (AgNPs), and platinum NPs (PtNPs), are characterized by a strong absorption of visible and near-infrared light. The surface plasmon resonance (SPR) phenomenon provides noble metal NPs with distinctive characteristics that are ideal for phototherapy^[172]^. In brief, NP illumination results in SPR, defined as “a collective oscillation of the metal-free electrons with respect to the NP lattice that is in resonance with the light field”^[172]^. Changes in the shape or morphology of metallic NPs can alter the localized surface plasmon resonance (LSPR)^[173]^.

The LSPR of metal oxide-based nanomaterials also depends on NP size and shape. The LSPR determines the photothermal effect of conductive metal oxides^[174]^. Importantly, the LSPR can be tuned solely through changes in the composition of metal oxide NPs^[174]^. These properties are particularly useful when small NPs are required, for example to ensure rapid renal clearance.

### Noble metal nanoparticles to treat multidrug resistant cancer

#### Gold nanoparticles

Anisotropic gold NPs of different sizes, morphologies (nanorods, nanostars, nanocages), and surface coatings have been used as NIR-responsive NPs^[175]^. All of these parameters affect NP performance in biological systems^[176,177]^. Gold nanorods (AuNRs) generate two plasmon bands^[177]^, and their anisotropic shape improves tumor accumulation when compared with gold nanospheres^[178]^. Gold nanostars (AuNSs) are especially useful for theranostics, because the electromagnetic field is markedly enhanced at “spikes”^[179]^. In addition, the sharp tips of AuNSs may cause physical damage to cellular structures and organelles^[180]^. Several properties distinguish gold nanocages (AuNCs) from other gold NPs^[181]^. Thus, AuNCs provide interior space for drug loading and release. At the same time, their mechanical properties are ideally suited for biological environments.

Several studies demonstrate that the photothermal effect of AuNPs can counteract MDR. AuNRs containing a mesoporous silica shell (Au@SiO_2_) loaded with doxorubicin re-sensitized drug-resistant “MCF-7/ADR” cells to drug treatment^[88]^. The hyperthermia generated by NIR irradiation of Au@SiO_2_ increased the abundance of trimeric HSF-1. The transcription factor HSF-1 is a well-established regulator of stress responses. Trimeric HSF-1 accumulated in nuclei, which ultimately reduced the abundance of NF-κB^[182]^. The combination of Au@SiO_2_ with NIR irradiation also diminished the levels of P-gp and mutant p53^[88]^. Taken together, NIR illumination of Au@SiO_2_ initiated multiple processes that led to elevated drug sensitivity and reduced viability of “MCF-7/ADR” cells^[88]^.

Independent studies confirmed the efficacy of AuNR-based applications in colorectal cancer cells^[102]^. This was accomplished with an AuNR nanocomposite containing poly-histidine and a mesoporous silica coat for doxorubicin loading. Poly-histidine binds the P-gp inhibitor D-α-tocopherol polyethylene glycol succinate (TPGS) in a pH-dependent fashion and enhances NP release from endo-/lysosomes. The composite NP was successful for combined chemo- and photothermal therapy (chemo-PTT) of drug-resistant colorectal cancer cells (SW620/Ad300) *in vitro* and in experimental mice^[102]^.

AuNSs have been used to produce subcellular PTT. These NPs functioned as a localized “nanoheater” and drug delivery vehicle^[111]^. The approach involved multiple AuNS modifications to ensure the proper NP distribution and drug delivery: (1) hyaluronic acid, (2) a mitochondrial targeting peptide, and (3) doxorubicin. Hyaluronic acid (HA) functions as active targeting ligand. HA binds the cell surface receptor CD44, which is often overexpressed in tumor cells^[140,141]^. Hyaluronidases degrade HA; these enzymes are often highly abundant in the TME^[142]^.

The hyaluronic acid coat stimulated CD44-dependent cellular uptake of AuNSs. Upon digestion of hyaluronic acid, the targeting peptide localized the AuNSs to mitochondria. NIR-induced local PTT disrupted mitochondrial function, which decreased ATP production. As a result, drug efflux through P-gp, a process requiring ATP hydrolysis, was inhibited. At the same time, doxorubicin impaired cell survival. The combination of PTT and chemotherapy diminished the viability of SCC-7 squamous cell carcinoma cells, but was less effective in reducing tumor growth in SCC-7 mice. Importantly, the AuNSs enhanced doxorubicin retention in “MCF-7/ADR” cells, and AuNS/NIR treatment increased the survival of mice harboring “MCF-7/ADR” tumors. Collectively, these experiments showed that the AuNS-based “nanoheater” together with NIR irradiation decreased tumor cell proliferation *in vitro* and in mouse models. This reduction was observed for cancer cells with and without MDR^[111]^.

AuNCs defeated MDR in “MCF-7/ADR” cells through a combination of PTT and PDT^[112]^. The hollow interior of AuNCs was loaded with doxorubicin, quercetin and the phase-change material tetradecanol. AuNC cellular uptake was stimulated through the coating with PEGylated biotin. NIR illumination triggered AuNC-mediated localized heating which melted tetradecanol. Tetradecanol melting led to doxorubicin and quercetin release, which was accompanied by NIR-induced ROS production (PDT). For the AuNCs tested, several processes contributed to the death of “MCF-7/ADR” cells. Together, quercetin, ROS, and ATP-depletion diminished P-gp-dependent efflux of doxorubicin. The ensuing intracellular accumulation of doxorubicin, combined with ROS increase and ATP-depletion, promoted “MCF-7/ADR” cell apoptosis.

#### Platinum nanoparticles

Platinum nanoparticles (PtNPs) are characterized by extraordinary stability at extreme temperature^[92]^; their thermoplasmonic properties are ideal for photothermal therapeutics. Fucoidan is a natural bioactive polymer that can induce tumor cell apoptosis^[183]^. With “MCF-7/ADR” cells as model system, the impact of PtNPs coated with fucoidan and loaded with doxorubicin was examined^[113]^. These PtNPs were applied in a trimodal combination therapy (bio-thermo-chemo: fucoidan-PTT-doxorubicin), which integrated platinum-mediated PTT with two anticancer agents. The three branches of the treatment regimen worked synergistically and disrupted the MDR of “MCF-7/ADR” cells *in vitro* and *in vivo*. The possible underlying molecular mechanisms were identified. In particular, PtNP-based tumor cell elimination was accompanied by reduced signaling through the PI3-kinase/Akt/mTOR pathway, a route crucial for cancer cell survival and proliferation^[184]^.

#### Palladium nanoparticles

Palladium NPs (PdNPs) exhibit high photothermal conversion efficiency and photostability, which makes them excellent tools for PTT^[163,164]^. A recent study tested tetrapod-shaped copper-palladium alloy NPs (TNPs) for chemo-PTT, a combination of chemotherapy and PTT^[114]^. The copper-palladium alloy offers several benefits, such as tunable photothermal properties and copper-induced autophagy. Autophagy represents a major route for the degradation of proteins and organelles. In the context of cancer, autophagy can be cytoprotective or cytotoxic^[185]^. Chloroquine and 3-methyl adenine are established inhibitors of autophagy^[185]^.

Using copper-palladium alloy NPs, several cellular models confirmed the importance of NP morphology and composition for biomedical applications^[114]^. As such, the photothermal conversion efficiency of tetrapod-shaped PdNPs was greatly enhanced when compared to a spherical PdNP counterpart. Moreover, the copper content of the alloy NPs determined their ability to induce pro-survival autophagy. These features were explored for the killing of HeLa, 4T1 and “MCF-7/ADR” cells, and contributing mechanisms were uncovered. Specifically, NIR irradiation of cells containing TNP-1, a TNP with high copper content, elevated mitochondrial ROS production. The increase in ROS then triggered pro-survival autophagy. Under these conditions, pharmacological inhibition (3-methyl adenine, chloroquine) or genetic ablation (*Atg5* knockout) of autophagy profoundly increased the cytotoxicity. The synergistic effect of TNP-1/NIR and the autophagy inhibitor 3-methyl adenine led to the efficient elimination of “MCF-7/ADR” cells. A synergistic effect of TNP-1-mediated PTT and autophagy inhibitors was also observed in “MCF-7/ADR” tumor-bearing NOD/SCID mice. Compared with control animals, PTT/autophagy inhibition decreased the tumor weight by ~86% (3-methyl adenine) or 93% (chloroquine). Together, these experiments uncovered the potential of combined PTT and autophagy modulation for the killing of MDR and other cancer cells *in vitro* and *in vivo.*

### Lanthanide-doped upconverting nanoparticles

Lanthanide-doped upconverting nanoparticles (UCNPs) absorb NIR light, which is converted via an anti-Stokes shift to light with higher energy. The light produced can be in the UV, visible, or NIR range of the spectrum^[165,186]^. UCNPs are especially interesting for biomedical research because their emission wavelength is tunable^[187]^. UCNPs have been evaluated for different modalities of cancer therapy, including the elimination of MDR cancer cells.

Several studies examined the effectiveness of UCNP-mediated PDT. To this end, different photosensitizers were conjugated to UCNPs and activated by NIR irradiation^[115,131]^. In one example, nanocomposites containing TiO_2_ as inorganic photosensitizer (NaYF4:Yb/Tm-TiO_2_) were conjugated to folate as a cellular targeting moiety and then loaded with doxorubicin^[115]^. Low pH (pH 6.0, pH 5.0) stimulated the release of doxorubicin, which was further increased by NIR irradiation. NIR light was also used for PDT, as it stimulated ROS generation by the photosensitizer TiO_2_. When compared with free doxorubicin, the nanocomposite/NIR treatment resulted in a significant loss of “MCF-7/ADR” cell viability *in vitro* as well as *in vivo* for “MCF-7/ADR” tumor-bearing mice^[115]^.

A multimodal UCNP nanoplatform was generated to overcome the cisplatin resistance of human ovarian cancer cells (A2780cisR)^[131]^. For this purpose, the UCNPs were conjugated to a platinum (IV) prodrug and functionalized with the photosensitizer Rose bengal (RB). These RB-Pt(IV)-UCNPs combined PDT with platinum-based chemotherapy. In brief, NIR irradiation of RB-Pt(IV)-UCNPs generated the visible light to activate Rose bengal and produce singlet oxygen (^1^O_2_). This led to an increase in cellular ROS levels, while delivery of the platinum (IV) prodrug facilitated genomic DNA crosslinking. The chemo-PDT combination treatment was toxic for cisplatin-sensitive (A2780) cells and their cisplatin-resistant counterparts (A2780cisR).

Another lanthanide-based approach was employed for combined chemo-PDT treatment^[126]^. Specifically, a lanthanide-doped silica framework containing europium and gadolinium oxides (Eu_2_O_3_, Gd_2_O_3_) was loaded with doxorubicin. NIR-irradiation of these EuGdOx@MSF-DOX NPs produced singlet oxygen, diminished P-gp levels, and released doxorubicin. The chemo-PDT strategy profoundly reduced the viability of “MCF-7/ADR” cells^[126]^.

Liposome/UCNP hybrid vesicles, called UCNP@Azo-Lipo/DOX, have been generated for the light-controlled release of doxorubicin^[123]^. The nanocarrier system is unique, as it addressed an important therapeutic issue - one-time burst *vs.* repeated cycles of drug delivery. UCNPs that emit UV and visible light upon NIR irradiation were key to control drug release and accomplish multiple delivery cycles.

Beginning with UCNPs, the delivery system was assembled in a stepwise fashion. A phospholipid monolayer was added to the UCNP surface to prevent particle agglomeration in aqueous environments. The UCNPs and doxorubicin were then co-encapsulated in artificial vesicles. The lipid bilayer of these vesicles contained azobenzene derivatives that undergo light-induced *cis/trans* isomerization^[188]^. UV light generates *cis*-isomers, which disrupts the lipid bilayer integrity and thereby promotes doxorubicin release from the vesicle. Visible light favors the formation of *trans-*isomers, which likely reseals the lipid bilayer. Using NIR-responsive UCNPs as the source of UV and visible light, the duration and intensity of NIR irradiation regulates drug release. Notably, multiple rounds of regulated drug release could be performed.

The system was tested in HeLa, MCF-7 and “MCF-7/ADR” cells *in vitro*; the combination of UCNP@Azo-Lipo/DOX and NIR treatment efficiently reduced the viability of all cell lines. Interestingly, UCNP@Azo-Lipo + NIR irradiation diminished the abundance of MRP1 (ABCC1) in “MCF-7/ADR” cells, which may re-sensitize cells to doxorubicin. Further evaluation of the delivery system was carried out with nude mice bearing MCF-7 or “MCF-7/ADR” derived tumors. (Folate was added to the lipid bilayer to improve tumor targeting.) Tumor growth was markedly reduced over a 2-week period, demonstrating that MDR could be overcome with the nanocarrier. Collectively, the study established a NIR-responsive delivery system that offers sophisticated control through repeated cycles of tunable drug release.

### Copper chalcogenides

The chalcogens, or oxygen family of elements, include oxygen, sulfur, selenium, tellurium, and polonium. Metal oxide-based NPs are discussed in a separate section (see below). Due to their tunable localized surface plasmon resonance, copper sulfide (Cu_2-x_S), copper selenide (Cu_2-x_Se), and copper telluride (Cu_2-x_Te) have favorable properties for NIR-based PTT and PDT^[166-170,189,190]^. In the context of medical applications, copper chalcogenides can resensitize chemoresistant tumor cells to drug treatment^[116,136]^. MDA-MB-453 breast cancer cells have high DNA methyltransferase (DNMT) activity and a mutation in the *PTEN* phosphatase gene; both features contribute to chemoresistance. However, a combinatorial approach based on doxorubicin-loaded Cu_2-x_Te nanocubes and NIR irradiation eliminated MDA-MB-453 cells *in vitro*^[136]^. With doxorubicin alone, the *in vitro* viability of MDA-MB-453 cells was largely unaffected (~90% survival). By contrast, cell viability decreased to 11% when combined chemo-PTT-PDT therapy was performed with doxorubicin-loaded Cu_2-x_Te nanocubes. Due to the intrinsic ability of Cu_2-x_Te nanocubes to increase ROS production upon NIR illumination, the system is independent of extrinsic photosensitizers.

Another study treated “MCF-7/ADR” cells with a multifunctional CuS-based nanostructure^[116]^. When irradiated with NIR light, the NPs released NO, and doxorubicin accumulated in cells. This was accompanied by a small decrease in P-gp abundance and a ~30% loss in the viability of “MCF-7/ADR” cells compared with untreated controls.

### Transition metal dichalcogenides

Two-dimensional transition metal dichalcogenides (2D TMDCs) are sandwich-like nanostructures, composed of a central layer of transition metal, surrounded by a layer of chalcogenides on each side ^[191,192]^. TMDCs are generally described as MX_2_, with M representing the transition metal (group 4-10 in the periodic table) and X the chalcogen (S, Se, or Te)^[193]^. TMDCs display strong NIR absorption, tunable optical properties, and a large surface area available for modification. Accordingly, TMDCs are suitable nanomaterials for PTT. When combined with conventional chemotherapy, the PTT or PDT elicited by various TMDCs (*e.g.*, MoS_2_^[194]^, MoSe_2_^[195-197]^, WS_2_^[198]^) efficiently eliminated tumor cells.

The TMDC-based multifunctional nanoplatform MoS_2_-PEI-HA^[117]^ carries hyaluronic acid (HA) for active targeting to the tumor site. To achieve localized drug release, the nanomaterial takes advantage of HA degradation by hyaluronidases. These enzymes are often highly abundant in tumors and the TME^[142,199]^. Combined with NIR irradiation, Dox@MoS_2_-PEI-HA (the doxorubicin-loaded version of MoS_2_-PEI-HA) significantly reduced “MCF-7/ADR” viability *in vitro* when compared with doxorubicin or Dox@MoS_2_-PEI-HA alone^[117]^. Several mechanisms likely contributed to “MCF-7/ADR” elimination, such as increased doxorubicin accumulation, heat production, and diminished P-gp abundance. The Dox@MoS_2_-PEI-HA/NIR combination was also effective for the treatment of “MCF-7/ADR” tumor-bearing mice. At day 25 of the experiment, the multimodal therapy prevented tumor growth by ~96%. Moreover, no tumor recurrence was observed, which was detected in all other experimental groups at day 14 or earlier.

### Metal oxides

Metal oxides have been widely studied in cancer research; specific examples include iron^[200-202]^, titanium^[203-205]^, and tungsten oxides^[206-208]^. Iron oxides are of therapeutic interest because they absorb the energy of the magnetic field to produce hyperthermia, which can be applied for the killing of cancer cells^[70,209,210]^.

The photothermal effect of metal oxides can also be used to release NO from a heat-sensitive NO donor (SNO)^[118]^. To this end, Fe_3_O_4_@polydopamine@mesoporous silica NPs were coupled to transferrin for cellular targeting and to SNO for NO production. These phototriggered NO nanogenerators (PTNGs) were loaded with doxorubicin and assessed in “MCF-7/ADR” cells *in vitro* and in “MCF-7/ADR” tumor-bearing BALB/c nude mice. Notably, when PTNGs were combined with NIR irradiation, the abundance of P-gp was significantly reduced in “MCF-7/ADR” cells. At the same time, the intracellular concentration of doxorubicin increased markedly. Moreover, the combination doxorubicin-loaded PTNGs/NIR profoundly diminished the viability of “MCF-7/ADR” cells when compared with doxorubicin-loaded PTNGs alone. These results support the idea that NO contributed to the therapeutic effects of PTNGs. *In vivo* studies confirmed this interpretation, as the combination treatment doxorubicin-loaded PTNGs/NIR led to a marked reduction of tumor volume and size in experimental mice.

To develop new tools for the elimination of drug-resistant cancer cells, iron/gold NPs were incorporated in porous silicon particles^[157]^. The nanocomposites were super-paramagnetic and could perform multiple cycles of photothermal conversion. Aside from NIR light, the NPs responded to additional external stimuli. For instance, application of an electric field improved the uptake of nanocomposites, both for 2D and spheroid cultures of “MCF-7/ADR” cells. The increase in cellular uptake may be explained by the formation of micro-aggregates or the accumulation of nanocomposites on the cell surface. The latter process is commonly employed for drug delivery or magnetofection^[211]^.

When nanocomposites were loaded with doxorubicin, drug release was stimulated by lowering the pH from 7.4 to 5.4; the release was further increased by NIR irradiation. The highest level of drug accumulation in nuclei was observed when “MCF-7/ADR” cells were exposed to doxorubicin-loaded nanocomposites, an external magnetic field, and NIR irradiation. By contrast, the combination free doxorubicin/external magnetic field/NIR was ineffective to concentrate the drug in nuclei. The same results were obtained for “MCF-7/ADR”-derived spheroids. In summary, the study developed an iron/gold-containing nanocomposite that responded to several external stimuli and provided a tool to concentrate doxorubicin in the nuclei of MDR cancer cells.

### Carbon-based nanoparticles

Carbon nanomaterials are noticeable for their structural diversity, optical features, high biocompatibility and -in general- low cytotoxicity^[212,213]^. Nevertheless, carbon nanomaterials can modulate the immune system; depending on the particle features, immune reactions were activated or suppressed^[214]^. Carbon nanotubes, carbon nanospheres, graphene, graphene oxides, and fullerenes represent major classes of carbon-based NPs. Some of these nanomaterials are NIR-responsive and characterized by high photothermal conversion^[215-217]^. Due to their unique properties, carbon-based NPs are suitable for PTT or PDT^[212,218,219]^. Carbon nanotubes are the most frequently used carbon-based NPs that have been thoroughly examined for drug delivery^[220]^. They are characterized by excellent mechanical properties, which combine high rigidity with good flexibility^[221]^. The elongated morphology of carbon nanotubes likely facilitates the translocation across biological membranes^[222]^. At the same time, this shape may play a part in the cytotoxicity of carbon nanotubes^[214]^. By contrast, carbon nanospheres are less likely to cause physical damage to living cells^[124]^. Graphene oxide has several advantages for biological applications; it is stable, yet flexible, dispersible in aqueous biological environments and easily amenable to modification^[213,221]^.

#### Carbon nanotubes

Single-walled carbon nanotubes offer high NIR absorbance and large surfaces for drug loading^[223]^. These attributes were explored to treat chemoresistant human leukemia K562R cells^[138]^. K562R cells have elevated levels of P-gp, which is located in the plasma membrane^[139]^. To improve targeting to K562R cells, the carbon nanotubes were functionalized with an antibody against P-gp. Indeed, the antibody enhanced binding of carbon nanotubes to K562R cells, but not to their drug-sensitive counterpart (K562S cells). To accomplish drug delivery, the single-walled carbon nanotubes were loaded with doxorubicin, which was efficiently released upon NIR irradiation. Taken together, the study showed that single-walled carbon nanotubes (conjugated to anti-P-gp antibodies and loaded with doxorubicin) combined with NIR irradiation significantly reduced the viability of K562R cells *in vitro.* Hence, functionalized single-walled carbon nanotubes can overcome MDR, which is ascribed - at least in part - to the increased biosynthesis of P-gp^[139]^.

To combat the MDR of “MCF-7/ADR” cells, single-walled nanotubes were modified with PEGylated phospholipids and loaded with doxorubicin^[125]^. Following cellular uptake of the carbon nanotubes and NIR illumination, doxorubicin accumulated in cell nuclei. This approach caused a significant loss of “MCF-7/ADR” cell viability when compared with non-irradiated control samples.

#### Carbon nanospheres

Hollow mesoporous carbon nanospheres can operate as drug carriers, while also providing PTT and PDT modalities. Together, these functionalities can combat the MDR of “MCF-7/ADR” cells^[124]^. Thus, NIR irradiation of doxorubicin-loaded carbon nanospheres initiated several processes that are relevant to cancer therapy. While heat, singlet oxygen, and ROS were produced, the intracellular concentration of doxorubicin increased. In addition, HSF-1 accumulated in nuclear foci, and the protein abundance of P-gp and p53 was reduced. Treatment with doxorubicin-loaded carbon nanospheres and NIR irradiation had synergistic effects, which cumulated in a significant loss of “MCF-7/ADR” cell viability *in vitro*.

#### Graphene oxides

The expression of several genes encoding drug efflux pumps is high in human MDA-MB-231 breast cancer cells^[134]^. To reduce the associated drug resistance, a chemo-PTT strategy with two therapeutic drugs was developed^[133]^. For this purpose, graphene was stabilized with poloxamer 188, a compound that also inhibits efflux pumps^[153]^. The nanocarrier was loaded with doxorubicin and irinotecan and then assessed for the killing of MDA-MB-231 cells. The dual drug-loaded graphene oxide increased the abundance of cyclin-dependent kinase inhibitors (p21, p27) and the tumor suppressor p53. These changes are consistent with an upregulation of cell apoptosis^[133]^. However, the effect may be independent of the nanocarrier, as the simultaneous incubation with doxorubicin and irinotecan led to the same changes in p21, p27 and p53 abundance. Nevertheless, the combination dual drug-loaded nanocarrier/NIR led to a profound reduction of tumor cell viability.

Another nanocarrier based on graphene oxide was developed for chemo-PTT of “MCF-7/ADR” cells^[119]^. NP surface engineering was aligned to the pH differences that NPs encounter during circulation, in the TME, and when located in cellular endosomes/lysosomes. Specifically, the graphene oxide carrier was coated with two different polymers. One of these polymers was further modified to provide pH-responsiveness. Following loading with doxorubicin, the pH-dependent changes of the nanocarrier had two effects that are pertinent to future *in vivo* applications. First, the overall positive charge of the delivery system at pH 6.8, which mimicked the TME, could facilitate uptake by tumor cells. Second, relevant to the lysosomal interior, doxorubicin release was stimulated at pH 5.0. Doxorubicin-loaded pH-responsive nanocarriers reduced the viability of “MCF-7/ADR” cells, which was further diminished by NIR irradiation. The effect of both treatment modalities was synergistic. Incubation with free doxorubicin or NIR irradiation alone led to only minor loss of cell viability. The two examples in this section demonstrate that the co-application of drug-loaded graphene oxide NPs and NIR light is suitable to overcome MDR in different *in vitro* models.

### Polydopamine

Due to their high photothermal conversion efficiency, polydopamine (PDA) and other polymers have been used as photothermal agents^[91]^. Polydopamine stands out because of its unique chemical properties, adherence to numerous surfaces, biocompatibility, high photoconversion rate, and ability to co-deliver multiple drugs^[224,225]^. Given their notable intrinsic PTT capability, a range of polydopamine-based structures (colloidal, hollow, and composite particles) have been assessed as NIR-responsive theranostic tools^[225]^. For example, polydopamine-containing nanostructures were examined in MDR cancer cells^[127-129]^.

Recent work exploited the polydopamine-mediated photothermal effect to release doxorubicin and generate NO. NO production was part of a triple treatment modality to surmount the MDR of “MCF-7/ADR” cells *in vitro* and *in vivo*. This was achieved with a polydopamine-based nanocomposite, which carried a NO donor and was loaded with doxorubicin^[127]^. NIR illumination triggered polydopamine-mediated PTT, NO and doxorubicin release. This triple combination of chemo-PTT and NO production diminished the viability of “MCF-7/ADR” cells and was associated with a marked reduction of P-gp abundance. Moreover, the triple combination strategy profoundly diminished the tumor volume in “MCF-7/ADR” tumor-bearing mice at treatment day 30.

Another study also applied a triple combination therapy to overcome MDR in “MCF-7/ADR” cells^[128]^. The approach used two different NIR-sensitive polydopamine NPs. One NP type could generate NO; the second was loaded with doxorubicin for chemo-PTT. Co-incubation with both NP classes and subsequent NIR irradiation caused a marked loss of “MCF-7/ADR” cell viability. The exposure to NO-producing NPs/NIR achieved important therapeutic goals that are relevant to MDR. Thus, the abundance of ABC efflux pumps was reduced. Simultaneously, intracellular ATP was depleted, which further diminished drug efflux.

The *in vitro* experiments were expanded to “MCF-7/ADR”-bearing mice. Successive treatment with (1) NO-producing NPs/NIR, and (2) doxorubicin-loaded NPs/NIR reduced the tumor volume profoundly. At the same time, caspase-3 abundance was increased, whereas Bcl-2 levels were reduced. These changes are consistent with increased tumor cell apoptosis in NP-treated animals when compared with control animals that received the vehicle PBS.

A polydopamine-based nanoplatform was also developed for chemo-PTT^[129]^. In particular, the microtubule-stabilizing drug docetaxel was loaded onto a biodegradable polymer, and then encapsulated by a polydopamine shell. The resulting NP was further modified with the P-gp inhibitor TPGS [Table 2]. The release of docetaxel from the nanoplatform was facilitated by lowering the pH from 7.4 to 5.0 and NIR irradiation. *In vitro*, the nanoplatform/NIR combination reduced the viability of “MCF-7/ADR” cells; the loss of viability was higher when compared with free docetaxel. In a mouse model, “MCF-7/ADR” tumor weight and volume were significantly decreased relative to the docetaxel-treated animals at day 14 of the treatment period. Collectively, the study demonstrated that polydopamine-based NPs are appropriate for chemo-PTT that delivers docetaxel and inhibits P-gp.

### Black phosphorus

The exploration of black phosphorus for biomedical applications, especially as an alternative to graphene-related materials, has begun only recently, and many hurdles remain^[226]^. Nevertheless, black phosphorus is relevant to cancer theranostics because it is biocompatible, biodegradable, and suitable for multiple applications. Examples are imaging, biosensing, drug delivery, PTT, PDT, sonotherapy, and the combination with other modalities, such as immunotherapy or cancer starvation therapy^[227-232]^. Key to the appeal of black phosphorus is its ability to form two-dimensional layered sheets. Importantly, the number of layers in a sheet can be controlled during fabrication. As a larger number of layers narrow the bandgap, the absorption properties of black phosphorus are tunable over the UV, visible, and NIR range of the light spectrum^[226,229,233]^. Although black phosphorus is biodegradable and degradation products are non-toxic, the material has notable disadvantages. Black phosphorous material has limited stability in the presence of oxygen or in aqueous solutions^[226]^. Nevertheless, capping layers or chemical surface modifications can protect black phosphorous nanosheets in biologically relevant environments. Moreover, black phosphorous nanosheets, in conjunction with NIR-irradiation, have been used to counteract the chemo-resistance of cancer cells^[120,121,132]^. This suggests that health-related applications based on black phosphorous nanomaterials are feasible in the future. Several recent publications support this optimism.

Polydopamine-coated black phosphorous nanosheets provided the platform for a combined gene/chemo-PTT approach to defeat cancer drug resistance^[120]^. The strategy was based on black phosphorous nanosheets that were functionalized and modified. Specifically, for P-gp gene knockdown siRNA was incorporated into the nanosheets, which were also loaded with doxorubicin. Association with the nanosheet protected P-gp siRNA against RNase-mediated degradation. Finally, a protective polydopamine film enhanced the stability and photothermal capacity of the nanosheets, and the DNA aptamer AS1411 was surface-conjugated for cancer cell targeting. The resulting nanosheets were assessed for gene/chemo-PTT.

Several parameters of the nanosheets controlled doxorubicin release, pH (discharge elevated at pH 5.0 as compared to pH 7.4), the polydopamine coat, and the NIR-triggered photothermal effect. Interestingly, NIR irradiation promoted the breakdown of the polydopamine coat and the black phosphorous nanosheet.

When tested in “MCF-7/ADR” cells, the highest toxicity was accomplished for the gene/chemo-PTT combination, whereas the simultaneous treatment with doxorubicin and P-gp siRNA had little impact. The effectiveness of the black phosphorous-based nanosheets was confirmed with “MCF-7/ADR” tumor-bearing nude mice *in vivo.* On treatment day 20, the targeted (AS411) gene/chemo-PTT approach led to a significant reduction of tumor volume relative to free doxorubicin/siRNA. Concurrently, P-gp abundance was markedly reduced in tumor cells.

A different study challenged cancer cell MDR with black phosphorous nanosheets that were designed for chemo-PTT-PDT. The three-pronged approach required NIR irradiation^[121]^. To this end, black phosphorous nanosheets were loaded with doxorubicin and protected by polydopamine. Finally, the nanosheets were modified with phenethyl isothiocyanate, a natural compound produced by cruciferous vegetables. Phenethyl isothiocyanate is relevant because it decreases the misfolding of mutant p53 protein^[234]^. (Note: the *TP53* gene is often mutated in tumor cells, and mutant p53 can promote cancer cell survival.) The chemo-PTT-PDT protocol was successful to overcome cancer cell MDR *in vitro* and *in vivo.* In particular, the triple combination treatment resulted in the lowest “MCF-7/ADR” cell viability when compared with control and other treatment groups. This outcome was verified *in vivo* with “MCF-7/ADR” tumor-bearing mice, as the smallest tumor volume was obtained with the chemo-PTT-PDT experimental group.

Another black phosphorous-based nanocarrier was examined with cisplatin-resistant human ovarian cancer cells (A2780cisR), hepatocellular carcinoma cells (HepG2cisR), their non-resistant counterparts (A2780cisN, HepG2cisN), and non-cancer cell lines^[132]^. For this study, the stability of black phosphorous nanosheets was improved by surface coordination with a cisplatin derivative. The PTT capability was preserved in these stabilized nanosheets. The nanosheets also elicited a DNA damage response in HepG2cisN and HepG2cisR cells, which was enhanced by NIR irradiation. Interestingly, the *in vitro* toxicity of the drug-modified black phosphorous nanosheets was higher for some cancer cell lines than for the non-tumor cell lines studied. In the future, these differences could potentially be exploited for the selective killing of tumor cells *in vivo*.

### Other NIR-responsive materials for the elimination of multidrug resistant cancer cells

This review presents NIR-responsive nanocarriers for the eradication of drug-resistant cancer cells. Our focus is on nanomaterials that are based on metals, carbon, polydopamine, or black phosphorus. Protocols that rely on other NIR-excitable agents are not discussed. However, it should be noted that light-responsive vesicles, micelles, and small molecule nanoassemblies continue to be assessed for cancer theranostics^[235-237]^.

## Limitations of NIR-responsive nanomaterials to defeat cancer drug resistance and future directions

**Figure 3:**
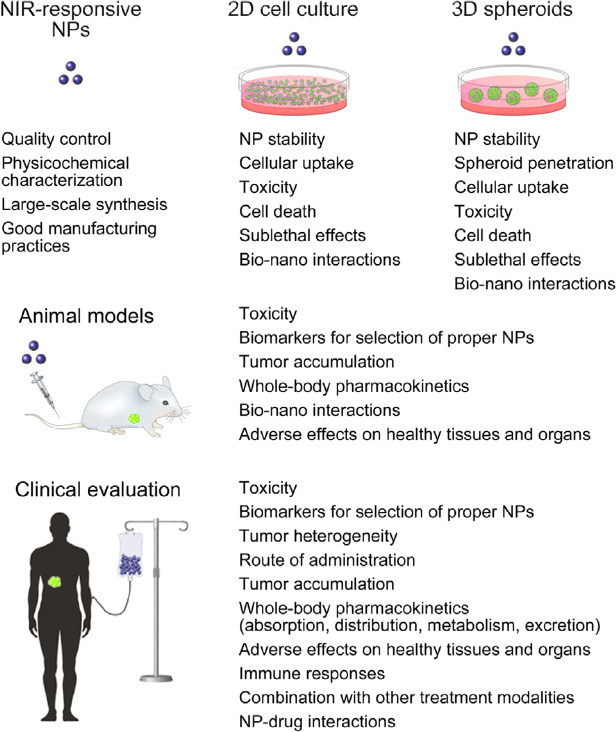
Important parameters for the evaluation of near-infrared (NIR)-responsive nanoparticles. The proper design, synthesis and evaluation of NIR-responsive nanoparticles (NPs) require methodical and quantitative assessment at different stages. Beginning with optimization of the synthesis, NPs are evaluated in 2D and 3D cell cultures, as well as in pre-clinical animal models^[253]^. If NPs perform adequately at these stages, they may progress to clinical trials. The figure depicts some of the critical readouts for each level of evaluation. See main text for additional details

Despite great promise of the NIR-responsive NPs discussed here, technical and other restrictions have so far prevented their wide-spread use for the improvement of human health^[51]^. The current shortcomings are related to diverse issues, ranging from NP synthesis to the optimal choice of molecular targets for NP-based therapies. To generate clinically relevant treatment regimens with NIR-responsive NPs in the future, multidisciplinary solutions are mandatory. They have to be accompanied by rigorous evaluation of NP properties and performance, from the initial synthesis to clinical trials [Figure 3]. Table 5 summarizes the topics that will benefit from further research and development.

**Table 5:** Factors and pathways involved in cancer MDR

Factor, pathway	Future studies related to stimuli-responsive NPs and multidrug resistant cancer
Long-term NP toxicity	Evaluation of NP toxicity after long-term exposure or repeated treatment cycles in appropriate *in vivo* models
NP clearance	Appropriate NP clearance, especially for multiple rounds of treatment
Combination therapy	Evaluation of efficacy for NP-based and other treatment modalities; whole body pharmacokinetics; assessment of synergistic effects for multi-modal therapy
Different drug transporters	Explore antibodies for NP targeting and transporter inhibition
Other factors promoting MDR	Gene mutations, epigenetic changes, cell signaling
Cancer stem cells (CSC)	Increase CSC vulnerability to NP-based treatment
Tumor microenvironment	Tumor vasculature, immune and other cells in TME, non-cellular components of TME
Immune system	Harness or enhance immune functions to eliminate MDR cancer cells
Stress resistance	NF-κB, HSF-1, molecular chaperones
Cancer cell and TME metabolism	Hypoxia, autophagy, other metabolic changes related to cancer cell survival
microRNAs	Regulation of tumor cell survival
Computational and mathematical modeling	Predict outcomes of NP-based regimens and test models *in vitro* and *in vivo*
Non-druggable targets	Silencing strategies, gene editing

Near-infrared (NIR)-responsive nanoparticles (NPs) impact various components and activities, which are often not fully characterized. Alternative and complementary strategies are proposed that may enhance the efficacy of NIR-responsive NPs for the treatment of chemo-resistant cancers. Additional details are provided in the main text. MDR: multidrug resistance; TME: tumor microenvironment

### Stimulus-responsive NPs

#### Synthesis and quality control

As discussed for theranostic nanomaterials in general^[57]^, the fabrication and quality control of NIR-responsive NPs are often complex, and large-scale synthesis is not a trivial task^[239,240]^. Yet, simple production protocols with low batch-to-batch variability and good manufacturing practices are necessary for the transition to clinical trials^[241]^. These features, and ideally low costs, are pre-requisites for health application and commercialization; they are frequently neglected in basic research.

#### Nanomaterial properties

Besides the constraints imposed by tumor location and vulnerability of healthy tissues, the nanomaterial properties also determine the effectiveness of NIR irradiation. In particular, the photoconversion efficiency of NPs has to be matched to the treatment modality. Thus, low quantum yield of upconverting nanomaterials will be inadequate for applications that rely on high photon upconversion^[242]^.

#### Adding new features to NIR-responsive NPs

Although simple and reliable protocols are compulsory for NP synthesis, new attributes could enhance their *in vivo* performance. In particular, relatively unexplored for the applications discussed here is the incorporation of phase-change materials. Recent studies demonstrate the potential of tetradecanol for cancer theranostics^[112,243,244]^ and warrant further assessment for NP-based approaches.

### NIR irradiation

#### Selection of optimal NIR wavelength

Specific aspects of NIR-responsive NPs deserve further attention to improve their performance related to cancer MDR. The advantage of NIR light over visible or UV light for tumor irradiation is clearly established. Nevertheless, the NIR penetration depth is still limited; it depends on the tumor location, light intensity and wavelength^[82]^. Hence, inadequate penetration depth may preclude efficient NIR-induced drug release at some tumor sites^[165]^. Compared to the widely examined NIR-I window (700-900 nm), the NIR-II window (1,300-1,700 nm) offers enhanced tissue penetration^[80]^. (Note: the demarcation of NIR windows varies among publications.) Accordingly, NIR-II-responsive nanomaterials may be superior for the detection of small primary tumors and metastases^[245,246]^.

#### Effects on healthy tissue

Although generally considered safe, high-intensity NIR light, prolonged or repeated cycles of NIR exposure may have negative impact on healthy cells. This includes tissue overheating, membrane re-organization, neural stimulation, mitochondrial ROS production, mitochondrial DNA damage, and changes in gene expression^[82,247,248]^. While these side effects could be tolerable for cancer theranostics, they may have to be considered if treatment regimens require repeated rounds of NIR irradiation.

### Nano-safety

A major concern related to *in vivo* applications of nanomaterials is long-term safety. This applies especially to nanocarriers that are not biodegradable, could leach toxic components, or accumulate in various tissues and organs. This concern is well documented for metal- and carbon-based NPs, as they may concentrate in the liver, lung, and spleen, with potentially deleterious consequences for organ functions^[249-251]^.

*In vitro* models often provide information on the toxicity and mechanism-of-action. Nonetheless, *in vivo* studies are required to define the organismal toxicity, whole body pharmacokinetics, and immune responses related to nanomaterials^[252]^ [Figure 3]. Rodents, especially tumor-bearing mice, are the most common experimental animals for this research. However, many aspects of mouse models have only limited clinical relevance; their specific shortcomings have been discussed previously^[57]^.

### Bio-nano interactions

The European Union has developed guidelines for the characterization and evaluation of UCNPs^[187]^. In addition, recommendations for the preclinical assessment of nanomaterials are available^[253]^. Many of these recommendations also apply to the NIR-responsive nanomaterials discussed here. Mathematical and computational modeling approaches could provide additional guidance to the field^[254]^.

A surprising lack of insight into even basic aspects of bio-nano interactions impedes progress in the field and holds back clinical translation. The complexities of these interactions are only emerging. For example, the role of NP elasticity for cellular uptake has only recently been demonstrated^[255]^ and remains to be explored for the treatment of chemo-resistant cancer.

Remarkably, the pharmacokinetics and pharmacodynamics of NP-based applications, especially as they relate to MDR, are frequently poorly defined^[28]^. This applies in particular to whole-body pharmacokinetics of the stimulus-responsive NPs discussed here. Many studies combine multiple treatment modalities and claim synergistic effects. However, an appropriate quantitative calculation of synergy is rarely provided in published work^[256]^. Furthermore, whether biocompatible doses of the NP/NIR combination surmount MDR often remains to be determined. A rigorous understanding of these parameters is compulsory to propel the field towards clinically relevant contributions.

Current limitations are further illustrated by the focus on strategies that target drug efflux pumps and employ NPs for the direct cancer cell killing. By contrast, the NP-based modulation of other targets and especially cancer support systems remain to be further explored (see below). Thus, several key issues require attention to uncover the full potential of NIR-responsive NPs for cancer therapy.

### New potential targets for NIR-responsive NPs

#### Diverse causes of cancer drug resistance

In the context of MDR, nanomedicine has largely focused on ABC transporters. However, multiple biological mechanisms advance cancer MDR in human patients. Inclusion of MDR-relevant factors that are mostly ignored will expand the potential of therapeutic NPs. The epigenetic machinery, such as DNA methyl transferases (see above), provides possible targets for further research^[257]^.

#### Cancer stem cells, the tumor microenvironment, and immune responses

CSCs and the TME are major contributors to MDR and cancer-related patient death^[2,4,10,11]^. So far, NIR-absorbing NPs have been evaluated predominantly for the killing of drug-resistant proliferating tumor cells. Thus, including CSCs as well as the cellular and non-cellular constituents of the TME will broaden the range of NP applications. This may involve the development of NPs that can prevent -or reverse- CSC dormancy.

In the TME, tumor macrophages, metalloproteases, and the tumor vasculature are potential targets to offset MDR. NIR-sensitive upconverting nanocrystals that reprogram tumor-associated macrophages or aim at metalloproteases exemplify this strategy^[258,259]^. Emerging mechanisms of NP extravasation from endothelial cells highlight the importance of matching NP properties to the biological constraints of the TME^[59,260]^. In this scenario, improving transcytosis across endothelial cells could increase NP concentrations in the tumor. As well, appropriate NP surface modifications could achieve this task^[260]^. Interestingly, PDT delivered by NIR-responsive UCNPs can augment the permeability of tumor blood vessels, thereby promoting NP extravasation into the tumor^[261]^. The same outcome is accomplished by impairing the endothelial barrier function with magnetic nanomaterials^[262,263]^.

There has been immense effort to identify -and prevent- the immune responses elicited by NPs^[264]^. Yet, alternative approaches are also being considered. In particular, NPs could modulate the activities of innate and adaptive immune systems towards the ultimate goal of eliminating cancer cells^[57,265,266]^. As NIR photo-immunotherapy can activate immune functions directed against cancer cells^[267]^, the modality seems especially suited to enhance the performance of NIR-responsive NPs.

#### Cancer cell metabolism

Cancer cells and the TME display prominent metabolic and signaling alterations^[268]^. These pathological changes, exemplified by hypoxia and the increased abundance of glutathione, foster treatment resistance^[269,270]^. The rewiring of cancer cell metabolism offers a plethora of therapeutic opportunities that can be addressed with NIR-responsive NPs.

#### Non-druggable contributors to cancer multidrug resistance

Until now, strategies to overcome cancer MDR with NIR-responsive NPs have largely disregarded the possibility to target non-druggable components. Thus, NPs that deliver siRNAs or oligonucleotides^[271]^ to cancer cells and the TME could open new opportunities for nanomedicine. Furthermore, gold nanoclusters or AuNPs have successfully delivered components of the CRISP/Cas9 system for gene editing to cancer cells^[272,273]^. Future studies will determine to which extent this emerging methodology is suitable to counteract cancer cell MDR.

### Clinical translation

#### Clinical trials

To date, only a small number of cancer-related clinical trials have been carried out with the nanomaterials described in this review. Indeed, the vast majority of NPs that are currently in clinical trials are liposome-based^[274]^. A notable exception is AuroLase therapy, which uses AuroShells®, NIR-responsive gold-silica nanoshells, for the thermo-ablation of tumor cells. AuroLase therapy has been evaluated in clinical trials for lung, head-and-neck, as well as prostate cancer^[275]^. Promising treatment outcomes have been reported for prostate cancer^[276]^. Moreover, several magnetic NPs are FDA-approved for magnetic hyperthermia, which can be enhanced by NIR irradiation^[209,277]^.

#### Future directions

The potential of NIR-responsive NPs for cancer therapy has been widely emphasized. However, their specific role in therapy is not always clear. Many cancer patients receive standard treatment that consists of surgery, radiotherapy, chemotherapy, immunotherapy or combinations thereof. Combination treatments that eradicate MDR cancer cells by targeting different resistance pathways are particularly efficient. Consequently, appropriate NP-based approaches must be selected to complement standard or other treatment regimens.

## Conclusion

Taken together, there has been remarkable advancement in the design, synthesis and evaluation of NIR-responsive nanomaterials *in vitro* and in animal models of cancer cell MDR. At the same time, translation to the clinic has been rare. To achieve this transition, future studies will have to answer a wide variety of questions to ensure the successful treatment of human patients (examples in Figure 3). The topics range from basic physiology, such as NP-triggered immune responses, to the development of appropriate treatment regimens, as exemplified by the route of NP administration and dosage. In the long run, the ability to improve patient survival and/or quality of life will determine the success of NIR-responsive NPs in the clinic.

## Declarations

### Copyright

© The Author(s) 2020.
